# Role of no table salt on hypertension and stroke based on large sample size from National Health and Nutrition Examination Survey database

**DOI:** 10.1186/s12889-022-13722-8

**Published:** 2022-07-05

**Authors:** Zongqin Li, Lan Hu, Xiaoxia Rong, Jun Luo, Xuejie Xu, Yonglong Zhao

**Affiliations:** 1 Department of Neurology, Sichuan Mianyang 404 Hospital, Mianyang, 621000 Sichuan China; 2 Department of Neurology, Affiliated Hospital of Southwest Medical University, Luzhou, 646000 Sichuan China; 3 Department of Operations Management Division, Sichuan Mianyang 404 Hospital, Mianyang, 621000 Sichuan China; 4 School of Pharmacy, and Engineering Research Center for the Development and Application of Ethnic Medicine and TCM (Ministry of Education), Guizhou Medical University, Guiyang, Guizhou, 550004 China

**Keywords:** Salt, Unsaturated fatty acid, Hypertension, Stroke, Association

## Abstract

**Background:**

To assess the associations between no table salt and hypertension or stroke.

**Methods:**

The data of 15,352 subjects were collected from National Health and Nutrition Examination Survey (NHANES) database. All subjects were divided into no hypertension or stroke group (*n* = 10,894), hypertension group (*n* = 5888), stroke group (*n* = 164) and hypertension and stroke group (*n* = 511). Univariate and multivariate logistic regression analysis was used to measure the associations of salt type used with hypertension and stroke and co-variables were respectively adjusted in different models.

**Results:**

After adjusting age and gender, other salt intake was associated with 1.88-fold risk of hypertension (OR = 1.88, 95%CI: 1.44–2.46) and no table salt was associated with 1.30-fold risk of hypertension (OR = 1.30, 95%CI: 1.15–1.47). After adjusting age, gender, race, BMI, PIR, marital status, CVDs, whether doctors’ told them to reduce salt, and diabetes, the risk of hypertension was 1.23-fold increase in no table salt group (OR = 1.23, 95%CI: 1.04–1.46). After the adjustment of age and gender, the risk of hypertension and stroke was 3.33-fold increase (OR = 3.33, 95%CI: 2.12–5.32) in other salt intake group and 1.43-fold increase (OR = 1.43, 95%CI:1.17–1.74) in no table salt group.

**Conclusion:**

Other salt intake or no table salt were associated with a higher risk of hypertension or hypertension and stroke.

**Supplementary Information:**

The online version contains supplementary material available at 10.1186/s12889-022-13722-8.

## Background

Hypertension is reported to be a major cause of premature deaths and a heavy burden of cardiovascular morbidity and mortality, which resulted in approximately 7.1 million deaths and an estimated cost of $48.6 billion ever year [1]. Hypertension is widely validated to increase the risk of developing cardiovascular diseases (CVD) such as coronary heart disease (CHD) and stroke [2, 3]. Hypertension has strong associations with atherosclerosis deposits blocking and narrowing brain arteries, which has become a major risk factor for the occurrence of stroke [4, 5]. Stroke is reported to be the leading cause of death in China and the second leading cause of death all through the world [6]. Stroke is also the main reason of long-term neurological disability in adults, which leads to a substantial economic burden to the society and a decreased quality of life in patients [7]. Considering the considerable amount of economic burden to the family and society, more effective health care planning and prevention of hypertension and stroke are necessary.

Currently, some researches proposed that lifestyle changes may have significant effects on blood pressure control [8]. As high salt intake was reported to be associated with the risk of hypertension and cardiovascular events, restricting dietary salt has been proposed to be a method for hypertension prevention [9, 10]. Salt reduction was considered to be an important dietary target for 2025 to reduce the mortality of main noncommunicable diseases by the World Health Organization (WHO) [11]. In recent years, low-salt diet or event no-salt diet was advocated to improve health and prevent some diseases [12]. Previously, a study reported that about 10% of the sodium intake comes from discretionary salt use including table salt and salt added while cooking, which can be controlled by individuals [13]. For some people, to control salt use in daily life indicated low salt or no salt indicate adding low or no salt at table, and some other people added lite salt or salt substitute at table. Lite salt or salt substitute refer to sodium chloride in traditional salt is partially replaced with potassium chloride or magnesium sulfate, which are considered as a strategy under consideration by several countries for lowing blood pressure [14]. At present, various studies have explored the associations between sodium intake and the risk of hypertension or stroke [3, 15]. High sodium intake was associated with increased risk of hypertension or stroke. People was advocated to reduced salt use. But for common people, monitoring the salt contributions of specific foods and food groups, differentiating the inherent and processing-added sodium content of foods or other dietary sources were difficult [13]. It is easier for controlling the discretionary salt use including table salt and salt added while cooking to achieve the sodium intake reduction. Previous studies were focused on the volume of salt intake with the risk of hypertension and stroke, whether the different types of salt used just at table or during cooking were associated with the risk of hypertension or stroke were still unclear. Thus, the associations of doesn’t add salt product or adding different varieties of salt at the table or during cooking in hypertension or stroke still needs investigation, which might provide a guide for salt use in common people in the prevention of hypertension stroke.

In the current study, the associations of different salt types added at table or no table salt with the occurrence of hypertension and stroke were assessed based on the data from National Health and Nutrition Examination Survey (NHANES) database.

## Methods

### Study population

**Fig. 1:**
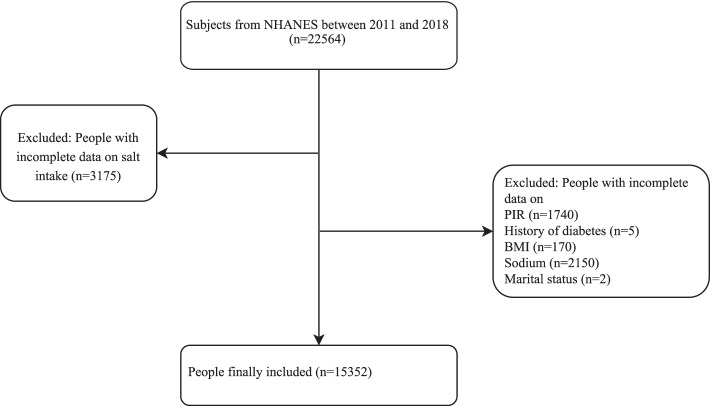
Screen process of participants in this study

The NHANES database included a multifaceted health examination on a nationally representative sample of the civilian, non-institutionalized population in the United States based on complex multistage stratified probability sampling methods [16]. NHANES data is publicly available and the data collection and data release were approved by the National Center for Health Statistics (NCHS) Ethics Review Board [17]. The current study collected the clinical data of 22,564 participants aged > 20 years from the NHANES database from 2011 to 2018. After excluding participants without data on salt intake, and baseline characteristics including poverty income ratio (PIR), history of diabetes, body mass index (BMI), sodium and marital status, 15,352 subjects were involved in. All subjects were divided into no hypertension or stroke group (*n* = 9297), hypertension group (*n* = 4661), stroke group (*n* = 340) and hypertension and stroke group (*n* = 1054). The detailed screen process was shown in Fig. 1.

### Main variables and outcome variables

The data of all participants were collected and analyzed including age, gender, BMI (kg/m^2^, < 18.5 kg/m^2^, 18.5–24.9 kg/m^2^, 25.0–29.9 kg/m^2^ or ≥ 30.0 kg/m^2^), race (Mexican American, other Hispanic, non-Hispanic White, non-Hispanic Black or other), education level (less than 9th grade, 9-11th grade, high school graduate, some college, or college graduate), marital status (married, widowed, divorced, separated, never married and living with partner), and PIR, history of diabetes, history of cardiovascular diseases (CVDs; congestive heart failure, coronary heart disease, angina/angina pectoris and heart attack), whether the participant’s doctor told them to reduce salt intake, type of table salt used (ordinary salt, other salt or no table salt).

### Data measurement and variable definition

In NHANES database, health questionnaires were collected from all subjects in their home, and physical, laboratory and anthropometric examinations were performed in Mobile Examination Centers (MEC) by well-trained health technicians following standardized procedures.

People with hypertension, stroke, or hypertension and stroke were measured as outcome variables. Hypertension was defined as self-reported physician diagnosis, mean systolic blood pressure ≥ 140 mmHg or mean diastolic blood pressure ≥ 90 mmHg or use of antihypertensive medication [18]. Data from the Medical Conditions Questionnaire (MCQ160f) were applied to identify stroke diagnosis. Participants who replied “Yes” to the question “Has a doctor or other health professional ever told you that you had stroke?” were identified as stroke survivors [19]. Those who replied to have difficulties causing by stroke problems based on the Physical Functioning Questionnaire (PFQ) (063A-063E) were also defined to have stroke [20].

Hypertension and stroke was defined according to the self-reported physician diagnosis of both hypertension and stroke.

No table salt referred to no salt used or added at the table and in food preparation in household. Ordinary salt includes regular iodized salt, sea salt and seasoning salts made with regular salt, indicating using or adding these kinds of salt at the table or while cooking. Other salt indicated the lite salt or salt substitute at the table or while cooking. Potassium intake were calculated from the in-person 24-h dietary recall interview which was administered by trained interviewers using the USDA automated multiple-pass method [21, 22].

All subjects were asked to list all food and beverages consumed in the 24-h period from midnight to midnight on the day before the interview. The NHANES calculates the nutrient intake from food and beverage data based on the USDA’s Food and Nutrient Database for Dietary Studies (FNDDS) [23]. The FNDDS uses food composition data from the USDA National Nutrient Database or Standard Reference [24]. At the end of the dietary recall, participants were asked questions about discretionary salt use. The questions are:

1. What type of salt do you usually add to food at the table? Would you say ordinary salt (includes regular iodized salt, sea salt, and seasoning salts made with regular salt), lite salt, salt substitute, don’t use or add salt at the table, other, don’t know?
2. How often do you add ordinary salt to food at the table? Would you say rarely, occasionally, very often, refused, don’t know?
3. How often is ordinary salt or seasoned salt added in cooking or preparing foods in your household? Is it never, rarely, occasionally, very often, or you don’t know?

Another variable including the data on salt use from the NHANES was DR2SKY with the questions of salt used at table yesterday? (Did {you/SP} add any salt to {your/her/his} food at the table yesterday? Salt includes ordinary or seasoned salt, lite salt, or a salt substitute.) and what type of salt was it? (Was it ordinary or seasoned salt, lite salt, or a salt substitute?).

The BMI in all people was divided into four groups: malnutrition group (< 18.5 kg/m^2^), normal weight group (18.5–24.9 kg/m^2^), overweight group (25.0–29.9 kg/m^2^) or obesity group (≥ 30.0 kg/m^2^).

### Statistical analysis

All statistical tests were conducted by two-sided test. The sample data were subjected to a weighted manner to all analyses to account for the cluster sample design, oversampling, poststratification, survey nonresponse and sampling frame, and the weights were taken from sdmvstra, sdmvpsu and wtmec2yr variables in the NHANES database [25]. The mobile examination center (MEC) exam weight (wtmec2yr variables) was applied for weighting. The variable name for the masked variance unit pseudo-stratum was sdmvstra and the variable name for the masked variance unit pseudo-primary sampling units (PSUs) was sdmvpsu. The weight of the survey enabled it to be extended to the civilian noninstitutionalized US population. Sampling errors were calculated to determining their statistical reliability [26]. The measurement data of normal distribution were described by Mean ± standard deviation (Mean ± SD), the independent sample t test was applied for comparisons between groups. The non-normal distributed data were expressed by M (Q_1_, Q_3_), and differences between groups were compared by the Mann–Whitney U rank sum test. The enumeration data were described as n (%) and comparison of different groups was performed by chi-square test. Logistic regression analysis was used to measure the associations of salt types with hypertension and stroke. The odds ratios (ORs) and confidence intervals (CIs) were employed for evaluating the reliability of an estimate. As for different types of salt intake, three models were established: Crude model: the model without adjustment; Model 1: adjusted for age and gender; Model 2: adjusted for age, gender, race, BMI and PIR. SAS 9.4 statistical analysis software was used for data analyzing. The svydesign and svyglm function in R package survey (4.02 version) was applied for logistic regression modeling and draw the forest plot. The detailed data analysis process was presented in Supplementary Fig. 1. *P* < 0.05 was considered as statistical difference.

## Results

### The characteristics of participants

In total, 22,564 subjects from NHANES between 2011 and 2018 were involved in this study. After excluding participants with incomplete data on salt intake (*n* = 3175) and subjects with incomplete data on PIR (*n* = 1740), history of diabetes (*n* = 5), BMI (*n* = 170), sodium (*n* = 2150) and marital status (*n* = 2), 15,352 subjects were finally included and were divided into no hypertension or stroke group (n = 9297), hypertension group (*n* = 4661), stroke group (*n* = 340) and hypertension and stroke group (*n* = 1054). Among them, 5062 people were aged 20–39 years, accounting for 32.97%, 5164 people aged 40–59 years, accounting for 33.64%; 4274 persons aged 60–79 years, accounting for 27.84% and 852 persons aged ≥ 80 years, accounting for 5.55%. There were 7302 (47.56%) males and 8050 (52.44%) females. The median PIR of all participants was 2.14. In the study population, 2144 (13.97%) people had diabetes, 12,795 (83.34%) people had no diabetes, and 413 (2.69%) people were in borderline. There were 5715 patients with hypertension, accounting for 37.23% and 1394 patients suffered from stroke, accounting for 9.08%. As for salt used, 9964 people used ordinary salt, accounting for 64.90%, 664 people used other salts (lite salt or salt substitute), accounting for 4.33% and 4724 people did not add salt at the table, accounting for 30.77% (Table 1).

**Table 1:** The baseline data of all participants in the study

Variables	Total (*n* = 15,352)
Age, n (%)	
20–39	5062 (32.97)
40–59	5164 (33.64)
60–79	4274 (27.84)
≥ 80	852 (5.55)
Gender, n (%)	
Male	7302 (47.56)
Female	8050 (52.44)
Race, n (%)	
Mexican American	1906 (12.42)
Other Hispanic	1493 (9.73)
Non-Hispanic White	6223 (40.54)
Non-Hispanic Black	3481 (22.67)
Other	2249 (14.65)
Education level, n (%)	
Less than 9th grade	1086 (7.07)
9-11th grade	1798 (11.71)
High school graduate	3440 (22.41)
Some college	4900 (31.92)
College graduate	4128 (26.89)
Marital Status, n (%)	
Married	7879 (51.32)
Widowed	1085 (7.07)
Divorced	1728 (11.26)
Separated	520 (3.39)
Never married	2858 (18.62)
Living with partner	1282 (8.35)
BMI (kg/m^2^), n (%)	
< 18.5	221 (1.44)
18.5–24.9	4055 (26.41)
25.0–29.9	4868 (31.71)
≥ 30.0	6208 (40.44)
PIR, M (Q_1_,Q_3_)	2.14 (1.11,4.12)
Diabetes, n (%)	
Yes	2144 (13.97)
No	12,795 (83.34)
Borderline	413 (2.69)
CVDs, n (%)	
Yes	14,011 (91.26)
No	1291 (8.41)
Unknown	50 (0.33)
Reduce salt, n (%)	
Yes	3158 (20.57)
No	8565 (55.79)
Unknown	3629 (23.64)
Hypertension, n (%)	
Yes	5715 (37.23)
No	9637 (62.77)
Stroke, n (%)	
Yes	13,958 (90.92)
No	1394 (9.08)
Type of table salt used, n (%)	
Ordinary salt	9964 (64.90)
Other Salt	664 (4.33)
No table salt	4724 (30.77)

*BMI* body mass index, *PIR* poverty income ratio, *CVDs* cardiovascular diseases

### Comparisons of characteristics of participants among different groups

All study population was divided into four groups, 9297 people were no hypertension or stroke, 4661 people were hypertension patients, 340 people were stroke patients, and 1054 people were hypertension and stroke patients, The results depicted that the proration of people in different age (χ^2^ = 3036.02, *P* < 0.001), race (χ^2^ = 354.30, *P* < 0.001), education level (χ^2^ = 361.77, *P* < 0.001), marital status (χ^2^ = 1041.96, *P* < 0.001), BMI (χ^2^ = 815.64, *P* < 0.001), PIR (χ^2^ = 260.78, *P* < 0.001), diabetes (χ^2^ = 1697.86, *P* < 0.001), CVDs, doctors told them to reduce salt and change type of table salt used (χ^2^ = 207.99, *P* < 0.001) groups among no hypertension or stroke, hypertension patients, hypertension and stroke patients were statistically different (Table 2).

**Table 2:** Comparisons of characteristics among different groups

Variables	No Hypertension or Stroke (*n* = 9297)	Hypertension (*n* = 4661)	Stroke (*n* = 340)	Hypertension and Stroke (*n* = 1054)	Statistic	*P*-value
Age (years), n (%)					χ^2^ = 3036.017	** < .001**
20–39	4352 (46.81)	628 (13.47)	47 (13.82)	35 (3.32)		
40–59	3182 (34.23)	1593 (34.18)	116 (34.12)	273 (25.90)		
60–79	1539 (16.55)	2022 (43.38)	135 (39.71)	578 (54.84)		
≥ 80	224 (2.41)	418 (8.97)	42 (12.35)	168 (15.94)		
Gender, n (%)					χ^2^ = 5.701	0.127
Male	4420 (47.54)	2256 (48.40)	158 (46.47)	468 (44.40)		
Female	4877 (52.46)	2405 (51.60)	182 (53.53)	586 (55.60)		
Race, n (%)					χ^2^ = 354.297	** < .001**
Mexican American	1317 (14.17)	450 (9.65)	40 (11.76)	99 (9.39)		
Other Hispanic	955 (10.27)	411 (8.82)	29 (8.53)	98 (9.30)		
Non-Hispanic White	3629 (39.03)	1937 (41.56)	183 (53.82)	474 (44.97)		
Non-Hispanic Black	1790 (19.25)	1359 (29.16)	52 (15.29)	280 (26.57)		
Other	1606 (17.27)	504 (10.81)	36 (10.59)	103 (9.77)		
Education level, n (%)					χ^2^ = 361.767	** < .001**
Less than 9th grade	556 (5.98)	352 (7.55)	38 (11.18)	140 (13.28)		
9-11th grade	982 (10.56)	567 (12.16)	67 (19.71)	182 (17.27)		
High school graduate	1950 (20.97)	1119 (24.01)	86 (25.29)	285 (27.04)		
Some college	2933 (31.55)	1549 (33.23)	89 (26.18)	329 (31.21)		
College graduate	2876 (30.93)	1074 (23.04)	60 (17.65)	118 (11.20)		
Marital Status, n (%)					χ^2^ = 1041.964	** < .001**
Married	4725 (50.82)	2555 (54.82)	159 (46.76)	440 (41.75)		
Widowed	325 (3.50)	508 (10.90)	44 (12.94)	208 (19.73)		
Divorced	866 (9.31)	607 (13.02)	55 (16.18)	200 (18.98)		
Separated	280 (3.01)	172 (3.69)	19 (5.59)	49 (4.65)		
Never married	2180 (23.45)	528 (11.33)	41 (12.06)	109 (10.34)		
Living with partner	921 (9.91)	291 (6.24)	22 (6.47)	48 (4.55)		
BMI (kg/m^2^), n (%)					χ^2^ = 815.638	** < .001**
< 18.5	176 (1.89)	34 (0.73)	3 (0.88)	8 (0.76)		
18.5–24.9	3059 (32.90)	745 (15.98)	82 (24.12)	169 (16.03)		
25.0–29.9	3016 (32.44)	1501 (32.20)	89 (26.18)	262 (24.86)		
≥ 30.0	3046 (32.76)	2381 (51.08)	166 (48.82)	615 (58.35)		
PIR, M (Q_1_,Q_3_)	2.27 (1.15,4.30)	2.20 (1.18,4.25)	1.35 (0.91,2.48)	1.42 (0.88,2.48)	χ^2^ = 260.784	** < .001**
Diabetes, n (%)					χ^2^ = 1697.862	** < .001**
Yes	556 (5.98)	1046 (22.44)	90 (26.47)	452 (42.88)		
No	8585 (92.34)	3414 (73.25)	240 (70.59)	556 (52.75)		
Borderline	156 (1.68)	201 (4.31)	10 (2.94)	46 (4.36)		
CVDs, n (%)					χ^2^ = 1478.690	** < .001**
Yes	9052 (97.36)	4058 (87.06)	269 (79.12)	632 (59.96)		
No	232 (2.50)	585 (12.55)	68 (20.00)	406 (38.52)		
Unknown	13 (0.14)	18 (0.39)	3 (0.88)	16 (1.52)		
Reduce salt, n (%)					χ^2^ = 2010.879	** < .001**
Yes	922 (9.92)	1712 (36.73)	64 (18.82)	460 (43.64)		
No	6251 (67.24)	1882 (40.38)	162 (47.65)	270 (25.62)		
Unknown	2124 (22.85)	1067 (22.89)	114 (33.53)	324 (30.74)		
Type of table salt used, n (%)					χ^2^ = 207.986	** < .001**
Ordinary Salt	6394 (68.77)	2755 (59.11)	239 (70.29)	576 (54.65)		
Other Salt	305 (3.28)	263 (5.64)	15 (4.41)	81 (7.69)		
No table salt	2598 (27.94)	1643 (35.25)	86 (25.29)	397 (37.67)		

*BMI* body mass index, *PIR* poverty income ratio, *CVDs* cardiovascular diseases

### Multivariate logistic regression analysis

**Fig. 2:**
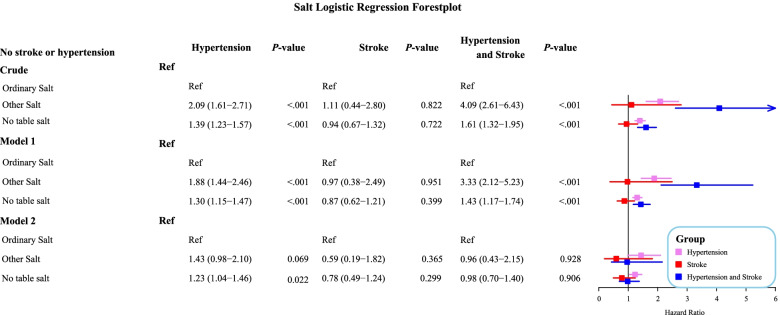
Forest plot of multivariable analysis of the associations between salt types and hypertension, stroke or hypertension companied with stroke

Compared with people consuming ordinary salt, the risk of hypertension was 2.09-fold increased (OR = 2.09, 95%CI: 1.61–2.71) in other salt intake group and 1.39-fold increased (OR = 1.39, 95%CI: 1.3–1.57) in no table salt group. After adjusting age and gender, other salt intake was associated with 1.88-fold risk of hypertension (OR = 1.88, 95%CI: 1.44–2.46) and no table salt was associated with 1.30-fold risk of hypertension (OR = 1.30, 95%CI: 1.15–1.47). After adjusting age, gender, race, BMI, PIR, marital status, CVDs, whether doctors’ told them to reduce salt and diabetes, the risk of hypertension was 1.23-fold increase in no table salt group (OR = 1.23, 95%CI: 1.04–1.46). Compared with those with ordinary salt at table, the risk of stroke in ordinary salt group or no table salt group was not statistically different (Fig. 2).

In comparison with people with ordinary salt at table, the risk of hypertension and stroke was 4.09-fold increase (OR = 4.09, 95%CI: 2.61–6.43) in other salt intake group and 1.61-fold increase (OR = 1.61, 95%CI: 1.32–1.95) in no table salt group. Post adjusting age and gender, the risk of hypertension and stroke was 3.33-fold increase (OR = 3.33, 95%CI: 2.12–5.32) in other salt intake group and 1.43-fold increase (OR = 1.43, 95%CI:1.17–1.74) in no table salt group. After adjusting age, gender, race, BMI, PIR, marital status, CVDs, whether doctors’ told them to reduce salt and diabetes, other salt intake and no table salt was not statistically associated with risk of hypertension and stroke (all *P* > 0.05) (Fig. 2).

## Discussion

In the present study, the data of 15,352 participants were collected from the NHANES database to analyze the associations of salt types added at table with hypertension and stroke. The result delineated that other salt intake or no table salt might be associated with an increased risk of hypertension. Other salt intake or no table salt might be also associated with an increased risk of hypertension and stroke. The findings of our study might give a reference for the use of salt at table in preventing the occurrence of hypertension and stroke and improving the prognosis of patients with hypertension or hypertension and stroke.

Currently, salt substitutes are used to replace ordinary salt (100% sodium chloride) where a portion of sodium is replaced with potassium chloride (usually 25%-30%) and/or magnesium sulphate (10%-14%). Several meta-analyses involving randomized controlled trials (RCTs) revealed that salt substitutes application decreased the systolic blood pressure and diastolic blood pressure in patients with hypertension [27]. Salt substitute might be an accessible and effective method for reducing the risk of death caused by stroke in patients with hypertension [28]. In this study, patients with other salt intake (lite salt or salt substitute) were associated with a higher risk of hypertension or hypertension and stroke. Some studies have indicated that people may prefer the taste of ordinary salt to salt substitutes and some people do not accept the taste of salt substitutes, so when they use salt substitutes, they might use more amount of salt, which actually resulted in a high sodium intake [29]. In addition, we found for people with more salt substitutes at table, the potassium intake was lower than those with ordinary salt intake (Supplementary Fig. 2). Previous studies have revealed that potassium is an essential nutrient and the addition of a high potassium diet could reduce the blood pressure in people [30, 31]. Also, some randomized controlled trials indicated that higher potassium intake could lower the blood pressure in those with hypertension [32]. Therefore, adequate potassium supplement was recommended in people especially hypertension people.

As for people do not add salt product at the table, excessive low salt diet might cause salt-sensitivity hypertension, as long-term low sodium intake might result in the high sensitivity to salt in human body and increased sodium intake might stimulate the secretions of hormones such as epinephrine and angiotensin, which led to hypertension [33]. Salt-sensitivity hypertension was a potential area requiring validation for further research, as some other researchers indicated that although a high-salt diet might increase the accumulation of sodium, the expansion of volume, and the adjustment of cardiac outputs, the autoregulation might maintain the flow via increasing the systemic vascular resistance, and causing the kidneys to excrete more salt and water, and therefore reducing systems to normal and minimizing the changes in blood pressure [34]. Another study also depicted that sodium reduction only decreased the blood pressure in participants with a blood pressure in the highest 25th percentile of all population and the author also suggested to reframe the policy of lowering dietary sodium intake in the general population and hypertension patients [35]. Sodium is main extracellular cation in the body to maintain intravascular volume, which is required in human body and salt restriction in humans may cause some adverse effects [36]. A previous study also reported that salt-deficient diet promoted cystogenesis in ARPKD via epithelial sodium channel [37]. Besides, people might intake more sodium rather than eat at table. Nowadays, commercial products infiltrate sodium insensibly into our nutrition and the involuntary sodium intake was high in daily life [38]. People who used other salt or do not add salt at table might prefer other commercial products with high sodium.

The findings of our study suggested that adding ordinary salt at table with appropriate volume is recommended for the prevention of hypertension. In addition, for people with hypertension or hypertension and stroke, adding lower volume of ordinary salt at table as well as enough potassium supplement were necessary for blood pressure control. This study measured the associations of salt with hypertension and stroke based on the data of 15,352 subjects from NHANES database. Our study involved in a large sample size and subgroup analysis was conducted in different types of salt, which might increase the reliability of our results. The findings of our study might provide a reference for the salt use at table or during cooking for common people. Several limitations existed in the current study. Firstly, the participants included in NHANES database were mainly from western countries, and whether the findings were suitable for people from oriental countries still needs validation in more studies. Secondly, the sample size in other salt group was small, which might decrease the statistical power. Thirdly, all data were collected from NHANES database, and important variables such as 24-h urine sodium of participants were not evaluated; the outcome variables were self-reported, which might cause bias. Fourthly, the self-reports of prior stroke in the NHANES stroke data represents a sample of stroke survivors, and we couldn’t analyze the data on those with acute stroke die, or are disabled to the extent that they are hospitalized, home-bound or institutionalized in long term care facilities. In the future, RCTs including large scale of sample size were required to verify the results in this study.

## Conclusions

This study analyzed the associations of different salt types and no table salt with hypertension and stroke based on the data of 15,352 subjects NHANES database. The results delineated that no table salt was associated with a higher risk of hypertension or hypertension and stroke. The findings suggested that salt intake is important and required in common people and in patients with hypertension or hypertension with stroke, necessary ordinary salt intake and enough potassium intake were required to control the blood pressure.

## Supplementary Information

**Additional file 1: Supplementary Figure 1.** The detailed process of data analysis in this study.

**Additional file 2: Supplementary Figure 2.** The potassium levels in people with different types of salt added at table.

## Data Availability

The datasets used and/or analyzed during the current study are available from the corresponding author on reasonable request. The original data were accessed from https://www.cdc.gov/nchs/nhanes/about_nhanes.

## Abbreviations

NHANES: National Health and Nutrition Examination Survey
BMI: Body mass index
CVD: Cardiovascular diseases
CHD: Coronary heart disease
WHO: World Health Organization
PIR: Poverty income ratio
MEC: Mobile Examination Centers
Mean ± SD: Mean ± standard deviation
OR: Odds ratio
CI: confidence intervals
