# Supplementary figures and images for: Role of no table salt on hypertension and stroke based on large sample size from National Health and Nutrition Examination Survey database
Source: BMC Public Health. 2022 Jul 5;22:1292. doi: 10.1186/s12889-022-13722-8 (PMC9254688; doi:10.1186/s12889-022-13722-8)

## The process of data analysis in this study

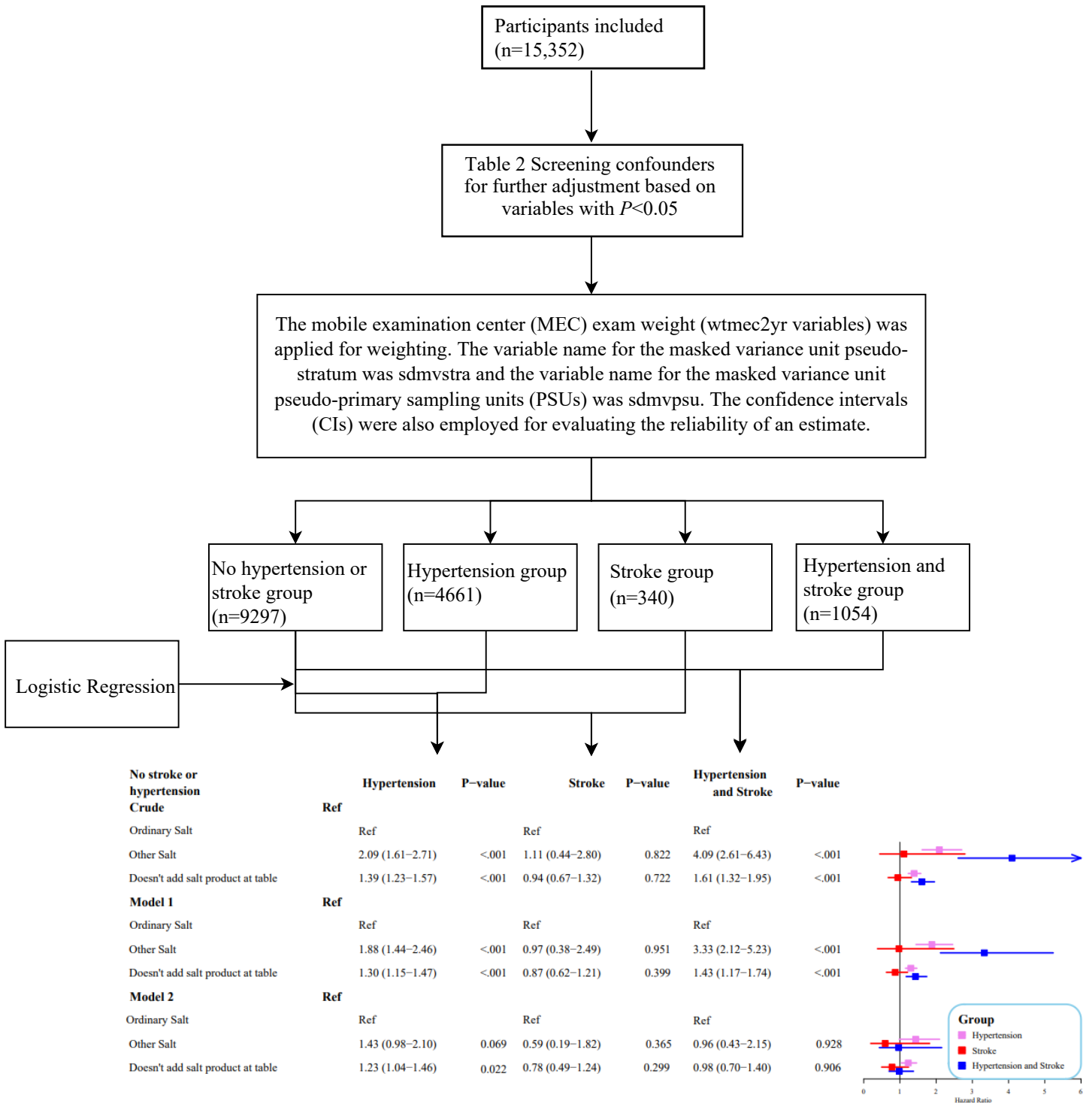

Supplement: Supplementary file 1 — Additional file 1: Supplementary Figure 1. The detailed process of data analysis in this study. [file 12889_2022_13722_MOESM1_ESM.pdf]

The potassium levels in people with different types of salt added at table

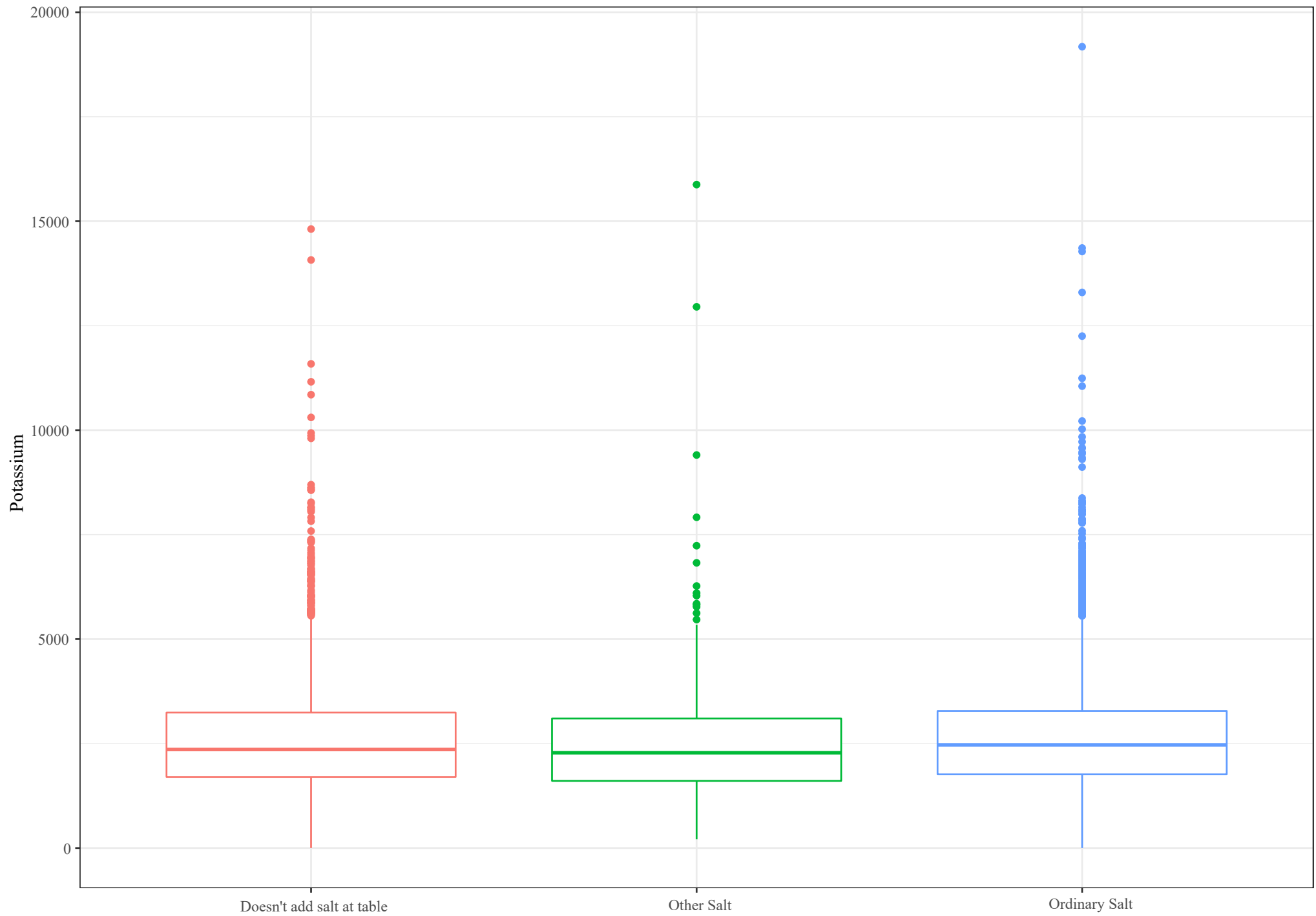

Supplement: Supplementary file 2 — Additional file 2: Supplementary Figure 2. The potassium levels in people with different types of salt added at table. [file 12889_2022_13722_MOESM2_ESM.pdf]
